# When embodiment matters most: a confirmatory study on VR priming in motor imagery brain-computer interfaces training

**DOI:** 10.3389/fnhum.2025.1681538

**Published:** 2025-09-25

**Authors:** Daniela Esteves, Katarina Vagaja, Alexandre Andrade, Athanasios Vourvopoulos

**Affiliations:** ^1^Institute for Systems and Robotics (ISR-Lisboa), Bioengineering Department, Instituto Superior Técnico, Lisbon, Portugal; ^2^Instituto de Biofísica e Engenharia Biomédica, Faculdade de Ciências da Universidade de Lisboa, Lisbon, Portugal

**Keywords:** sense of embodiment, virtual reality, motor imagery, brain-computer interfaces, event-related desynchronization

## Abstract

**Background:**

Virtual Reality (VR) feedback is increasingly integrated into Brain-Computer Interface (BCI) applications, enhancing the Sense of Embodiment (SoE) toward virtual avatars and fostering more vivid motor imagery (MI). VR-based MI-BCIs hold promise for motor rehabilitation, but their effectiveness depends on neurofeedback quality. Although SoE may enhance MI training, its role as a priming strategy prior to VR-BCI has not been systematically examined, as prior work assessed embodiment only after interaction. This study investigates whether embodiment priming influences MI-BCI outcomes, focusing on event-related desynchronization (ERD) and BCI performance.

**Methods:**

Using a within-subject design, we combined data from a pilot study with an extended experiment, yielding 39 participants. Each completed an embodiment induction phase followed by MI training with EEG recordings. ERD and lateralization indices were analyzed across conditions to test the effect of prior embodiment.

**Results:**

Embodiment induction reliably increased SoE, yet no significant ERD differences were found between embodied and control conditions. However, lateralization indices showed greater variability in the embodied condition, suggesting individual differences in integrating embodied feedback.

**Conclusion:**

Overall, findings indicate that real-time VR-based feedback during training, rather than prior embodiment, is the main driver of MI-BCI performance improvements. These results corroborate earlier findings that real-time rendering of embodied feedback during MI-BCI training constitutes the primary mechanism supporting performance gains, while highlighting the complex role of embodiment in VR-based MI-BCIs.

## 1 Introduction

Virtual Reality (VR) has emerged as a powerful tool for enhancing Brain-Computer Interface (BCI) applications, particularly in the domain of motor imagery (MI) training. MI-BCIs enable volitional and direct brain-to-device communication by bypassing conventional neuromuscular pathways, allowing users to control external devices through the mental rehearsal of movement without actual muscle activation (Pfurtscheller and Neuper, 2001). When integrated with VR, these systems benefit from immersive and ecologically valid interactive environments, which have been shown to enhance the Sense of Embodiment (SoE), allowing users to perceive a virtual avatar as part of their own body (Kilteni et al., 2012; Botvinick and Cohen, 1998; Lenggenhager et al., 2007; Petkova and Ehrsson, 2008). This embodiment illusion plays a crucial role in engaging users more effectively and influencing neural activity patterns (Esteves et al., 2025), making VR an ideal medium for neurorehabilitation and BCI training (Vourvopoulos et al., 2022; Batista et al., 2024; Škola and Liarokapis, 2018).

SoE arises from the integration of multiple sensory and cognitive cues, including visuomotor, visuotactile, and proprioceptive feedback (Guy et al., 2023). When these cues are congruent, users experience a heightened sense of ownership over the virtual body, along with an increased sense of agency—the feeling of control over avatar movements (Jeong and Kim, 2021). Research has demonstrated that inducing SoE through VR enhances engagement, immersion, and neurophysiological responses, making it a powerful tool for MI-BCI training (Petkova and Ehrsson, 2008; Slater et al., 2008).

VR-based MI-BCIs provide an integrated system where neurophysiological data recorded through electroencephalography (EEG) drive real-time interactions in immersive virtual environments. By leveraging multimodal sensory feedback, such as: visual; auditory; and haptic stimulation, VR has been shown to enhance MI performance, engagement, and neuroplasticity (Wang et al., 2019; Choi et al., 2020b). In particular, the effectiveness of VR-enhanced MI training is supported by its ability to strengthen sensorimotor activity, as measured by event-related desynchronization (ERD) in the Alpha and Beta frequency bands (Chen et al., 2023; Pichiorri et al., 2015; Vourvopoulos et al., 2016). Stronger ERD is associated with more effective motor learning, making VR-based MI-BCI systems promising for stroke rehabilitation and other motor impairments (Vourvopoulos et al., 2016; Choi et al., 2020a; Wen et al., 2021).

While VR has demonstrated clear benefits in MI-BCI training, for instance studies show that motor priming in VR can enhance ERD and improve BCI control (Vourvopoulos and Badia, 2016; Amini Gougeh and Falk, 2023), its potential as a preparatory mechanism suggests that sensorimotor engagement before training may facilitate subsequent MI performance. Nonetheless, the role of prior virtual embodiment (embodiment priming) in modulating MI-related EEG activity remains an open question. This priming familiarization with the virtual body by allowing participants to explore the virtual environment from a first-person embodied perspective, may augment the embodiment effect during training. Still, to our knowledge, only the study from Vagaja et al. (2024) has directly addressed this question, comparing MI-BCI performance with and without prior embodiment exposure. In that pilot work, we found no significant advantage of prior embodied over MI conditions, however, we noted important limitations, particularly the relatively small sample size and the use of a between-subject design, which may have introduced inter-subject variability. Thus, a gap remains in the literature regarding the potential effects of inducing embodiment in the virtual environment before MI training.

To address these, the present study builds upon our prior research (Vagaja et al., 2024) by implementing a within-subject design with an expanded sample size. This methodological improvement minimizes variability in individual MI responses, allowing for a more precise assessment of SoE's impact on MI-BCI training. By analyzing ERD patterns and lateralization indices under different embodiment conditions, this study aims to elucidate the extent to which prior virtual embodiment influences MI performance.

We hypothesize that exposure to an embodied virtual scenario prior to MI training will lead to stronger ERD responses, due to enhance users' ability to feel embodied during the MI task itself. Nonetheless, given the highly individual nature of embodiment experiences and the previous results of Vagaja et al. (2024)'s work, we also expect that this effect will depend on the subject personal ability to immerse themselves in virtual scenarios, feeling embodiment and ability to modulate their brain activity. Therefore, we anticipate a general trend toward stronger ERD when prior embodiment is present, but with inter-subject variability potentially moderating the effect.

Understanding the underlying neural mechanisms associated with SoE in VR-BCI applications will help to the expansion of theoretical models of embodiment and immersion in virtual scenarios, particularly in how it affects the brain activity, as well as contribute to the optimization of neurorehabilitation strategies and the development of more effective personalized training protocols. If prior embodiment can reliably enhance ERD induction during MI tasks, this would support the integration of a priming phase in MI-BCI training protocols for neurorehabilitation, for example, leading to improved engagement, and training outcomes, increasing the chances of neuroplastic changes. Furthermore, this study also elaborates on possible individual effects of embodiment experiences, contributing to personalized neurofeedback approaches.

## 2 Related work

Previous research has investigated the relationship between SoE and MI-induced brain activity. For instance, Evans and Blanke (2013) demonstrated that Virtual Hand Illusions (VHIs) and hand MI tasks share similar electrophysiological correlates, specifically ERD in frontoparietal brain areas, suggesting that SoE can enhance ERD patterns during MI training. Building on this, several studies have investigated the potential benefits of embodiment feedback in MI-based training. For example, Song and Kim (2019) demonstrated that an RHI-based paradigm with a motorized rubber hand significantly amplified MI-induced ERD.

The advent of VR has further expanded this line of research by enabling the replacement of a user's real body with a responsive virtual avatar, providing visual and proprioceptive feedback and facilitating VHI (Khademi et al., 2023; Lotte et al., 2013; Lenggenhager et al., 2007; Petkova and Ehrsson, 2008; Guy et al., 2023). Integration with BCI systems, as shown by Pérez-Marcos et al. (2009), demonstrates that SoE can be induced even in real-time online BCI paradigms, moving research from traditional non-digital RHI settings into fully immersive VR environments.

Multiple studies have confirmed that VR-induced SoE feedback positively modulates brain activity during MI tasks, enhancing motor learning and BCI control (Batista et al., 2024; Juliano et al., 2020; Škola and Liarokapis, 2018, 2022; Jeong and Kim, 2021). For example, Vourvopoulos et al. (2022) demonstrated that vibrotactile feedback combined with embodied VR led to stronger and more lateralized Alpha ERD compared to conventional 2D screen-based MI training. Similarly, Du et al. (2021) found that visuotactile stimulation of a virtual hand preceding an MI task resulted in greater ERD compared to stimulation of a rubber hand. However, these studies did not directly quantify embodiment or analyze their relationship with MI-induced ERD. Other studies examining this relationship reported inconsistent findings. While Braun et al. (2016) observed positive correlations, Škola and Liarokapis (2018) found no association, Škola et al. (2019) reported positive correlations for SoO but negative correlations for SoA, and Nierula et al. (2021) reported the opposite effects.

Overall, the literature supports the use of VR-embodied feedback to enhance ERD during MI-BCI training, particularly by inducing SoE during the MI task. This is consistent with the growing use of VR-based MI-BCI systems for neurorehabilitation, as MI stimulates lesioned sensorimotor areas, promoting neuroplasticity and supporting motor recovery (Daly and Huggins, 2015). By providing real-time embodied feedback, these systems enhance the effects of MI and facilitate brain activity modulation, thereby accelerating rehabilitation (Pichiorri et al., 2015; Vourvopoulos et al., 2016, 2019; Choi et al., 2020b). However, the optimal settings for embodiment induction remain unclear. For instance, Choi et al. (2020a) showed that head-mounted VR feedback improved MI-BCI performance by increasing immersion, presence, and cortical activation compared to non-immersive feedback, whereas Jeong and Kim (2021) found that VHIs and RHI conditions produced comparable ERD patterns. Individual differences in embodiment strength further complicate these results, as users may experience SoE differently even under identical VR conditions (Guy et al., 2023).

Most studies have focused on inducing embodiment during MI tasks and have assessed SoE only after VR training, rather than exploring its role in preparatory scenarios. To date, only Vagaja et al. (2024) has investigated whether inducing embodiment prior to MI training (priming) can enhance MI-related ERD, reporting no significant increase in ERD amplitude, no changes in ERD lateralization, and no improvement in BCI performance. Consequently, a substantial gap remains in understanding the optimal procedures for VR-embodied feedback to enhance ERD during MI training, as well as the potential benefits of prior embodiment exposure.

## 3 Methods

This study builds on data from a previously conducted Pilot study (Study 1), integrating it with newly collected data from an Extended study (Study 2), conducted with improved protocol and increased sample size. The resulting Combined Dataset includes a total of 39 participants. To maintain methodological consistency, identical procedures and analytical methods were applied across both datasets.

### 3.1 Study 1: pilot

The dataset from the Pilot study^1^ included 26 right-handed healthy participants (10 males, 16 females) randomly assigned to either the Control (five males, eight females) or Embodied (six males, seven females) group, with a mean age of 24.12 ± 5.99 years. More demographic information can be found in the original paper (Vagaja et al., 2024).

The experiment took place in a Virtual Environment (VE) resembling the real physical room, where participants viewed a gender-matched avatar seated at a desk with a virtual mirror above it. The VE was created using the Unity 3D engine, with avatars generated via Ready Player Me. Immersive feedback was delivered through an Oculus Rift CV1 headset, Oculus Touch controllers, and Constellation sensors for hand tracking. Furthermore, EEG signals were recorded using a wearable LiveAmp EEG amplifier (Brain Products GmbH) with 32 active electrodes placed according to the 10–20 system, sampled at 500 Hz.

Data collection consisted of three phases: (1) resting-state EEG recording (4 min), (2) embodiment phase in VR (5 min, induction or disruption), and (3) MI training in a similar VR scenario (15 min). During the embodiment phase, group conditions differed, in a between-subject design. The Embodied group experienced VR-induced SoE through visuomotor, visuotactile, and visuoproprioceptive triggers. For 3 min, they explored the VE from a first-person perspective, with their avatar synchronizing with their movements. This was followed by a 2-min VHI, where a virtual brush stroked their right virtual hand while they felt the corresponding real-hand stimulation. In contrast, the Control group experienced the same phase, but with disrupted triggers to break the illusion, specifically, viewing a third-person avatar moving independently and receiving incongruent brush strokes on the opposite hand during the VHI. A video illustrating the experimental phases is also available online^2^. Afterward, all participants completed a validated SoE and physical presence questionnaire using a 7-point Likert scale, adapted from Peck and Gonzalez-Franco (2021) and the Multimodal Presence Scale (MPS) (Makransky et al., 2017). They then performed a hand-grasp MI training task in the same VE from a first-person perspective. Training included 40 trials (20 per hand, randomly presented), each consisting of a 10-second resting period, followed by a 10-second MI period where a visual cue (arrow) indicated which hand to imagine grasping while observing the corresponding virtual hand move.

### 3.2 Study 2: extended

**Figure 1:**
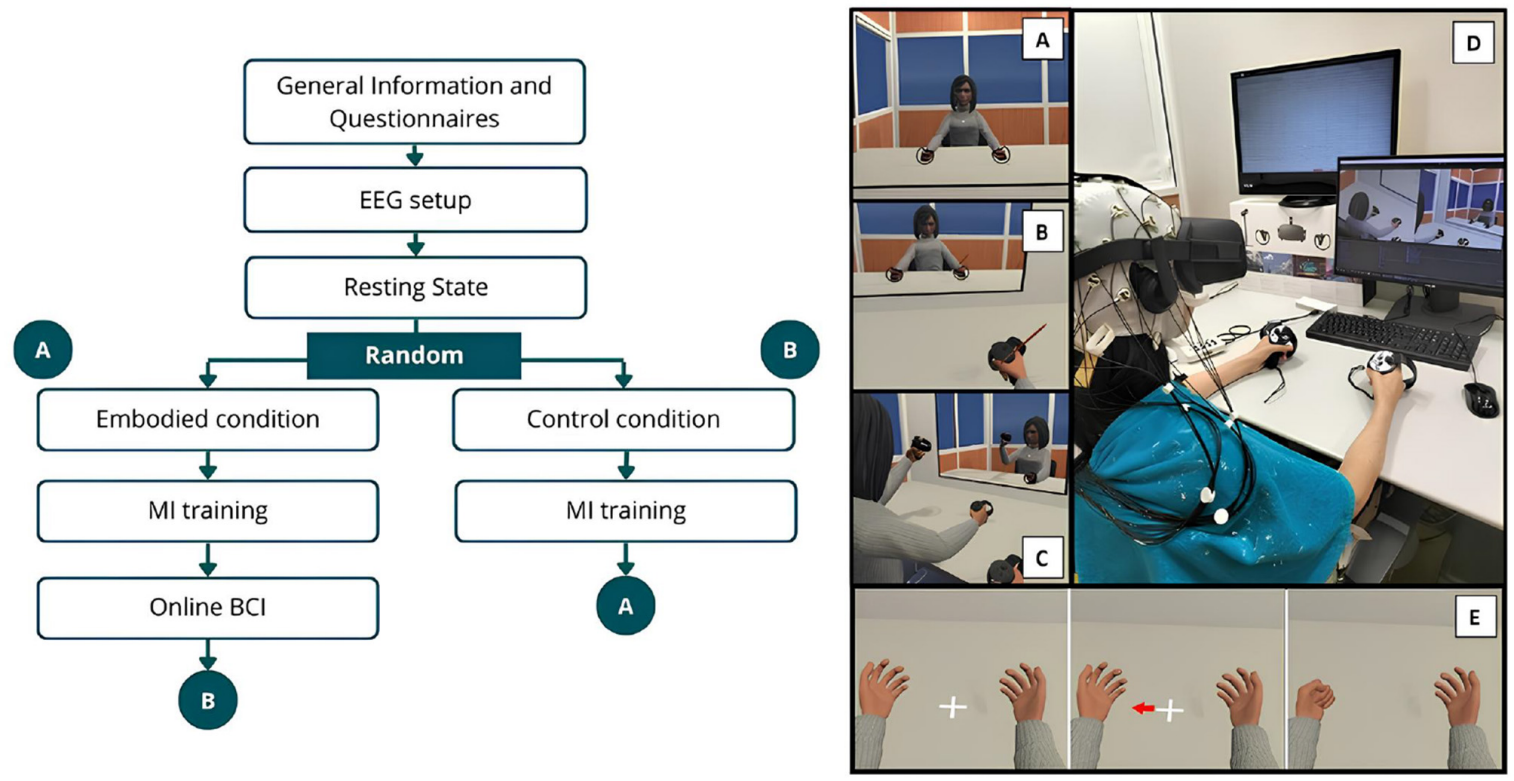
Schematic representation of the experimental design and VE. The left side illustrates the procedural sequence, while the right depicts the VE, where **(A)** Shows the VE and virtual avatar during the exploration phase of the Embodied condition, while **(B)** represents the brushing phase (VHI) of the same condition. **(C)** Represents the VE during the exploration phase of the Control condition. **(D)** Shows a female participant during the Control condition. **(E)** Illustrates the MI trial sequence, beginning with the resting period followed by the visual cue (arrow) indicating the start of the hand-grasping action.

The data collection procedure of the extended study followed the same approach as the Pilot study (Vagaja et al., 2024) but implemented a within-subject design. Figure 1 illustrates the procedure, outlining the data collection phases and the VE used.

#### 3.2.1 Participants

Only adults aged between 18 and 75 years, with a minimum of 9 years of schooling, and who do not suffer from severe neurological or psychiatric illness (defined as preventing participation in working life at the time of the study) were considered for the experiment.

A total of 15 participants were initially recruited for this study; however, two were subsequently excluded due to issues encountered during EEG recordings, specifically extensive artifacts or malfunctioning electrodes. Therefore, the newly Extended Dataset comprised data from 13 healthy subjects (seven females, 53.85%; 6 males, 46.15%), with a mean age of 26.08 ± 6.57 years and little to no prior experience with MI tasks. articipants completed the Vividness of Movement Imagery Questionnaire-2 (VMIQ-2) (Roberts et al., 2008) before the experiment, revealing low imagery ability (internal visual imagery: 1.711 ± 0.582; kinaesthetic imagery: 1.928 ± 0.840; external visual imagery: 2.111 ± 0.745, on a 5-point Likert scale). The Edinburgh Handedness Inventory (EHI) (Oldfield, 1971) confirmed all were right-handed with an average laterality quotient (LQ) of 62.05 ± 21.74, and all signed an informed consent following the ethical guidelines of the 1964 Declaration of Helsinki.

#### 3.2.2 Experimental design

The experiment consisted of seven recording phases, beginning with information and demographic questionnaires, followed by the EEG setup and a resting-state EEG recording. The VR headset was then carefully positioned over the electrodes and remained in place throughout the experiment to prevent displacement. Participants then proceeded to either the “Embodied" or “Control" condition in a randomized order. Each of these conditions concluded with the completion of a SoE and presence questionnaire before moving on to the hand-grasping MI training phase. After finishing one condition and its corresponding MI training, participants transitioned to the next, ensuring that each individual experienced MI training after Embodied (MI Embodied condition) and MI training after Control (MI Control condition; Figure 1). Additionally, after the MI training associated with the Embodied condition, participants completed two online BCI phases in a randomized order.

##### 3.2.2.1 Information and EEG setup

Participants were provided with a consent form, relevant study information, the EHI questionnaire, and a demographic questionnaire. The EEG setup was subsequently carried out using conductive gel to maintain electrode impedance below 10 kΩ.

##### 3.2.2.2 Resting state

This phase consisted of 2 min of eyes-open followed by 2 min of eyes-closed resting-state EEG recording, totaling 4 min.

##### 3.2.2.3 Embodied condition

Participants underwent a VR-induced SoE using visuomotor, visuotactile, and visuoproprioceptive cues. The phase began with instructions before participants entered the VE, where they viewed a gender-matched avatar from a first-person perspective (visuoproprioceptive trigger; Figure 1A). They then explored the VE for 3 min, looking around, seeing their reflection in the mirror upon the table, and moving their virtual hands, head, and torso while remaining seated. The avatar's movements were synchronized with their own (visuomotor trigger; Figure 1C). Following this, participants remained still and the VHI was implemented. They focused on their right hand as a virtual brush appeared, stroking the virtual hand for 2 min in perfect sync with the experimenter brushing their real hand (visuotactile trigger; Figure 1B). Afterward, participants exited the VE, resulting in a 5-min EEG recording, and verbally responded to the embodiment questionnaire.

##### 3.2.2.4 Control condition

This condition mirrored the Embodied condition but with disrupted embodiment cues. Participants entered the VE and viewed a gender-matched avatar from a third-person perspective (incongruent visuoproprioceptive trigger). The avatar's movements were independent of their real movements (incongruent visuomotor trigger). They then explored the VE for 3 min, followed by 2 min of disrupted VHI. Participants focused on their right virtual hand for a disrupted VHI, while felt their real hand being brushed, without visual confirmation in the VE (incongruent visuotactile trigger). After 2 min, the brushing stopped, participants exited the VE, and they verbally responded to the same embodiment questionnaire. Thus, by systematically introducing incongruence across visuoproprioceptive, visuomotor, and visuotactile triggers, this condition controlled for multisensory congruency effects and ensure that embodiment could not emerge, serving as a disembodied/control condition.

##### 3.2.2.5 MI training

This phase began with instructions explaining to the participant how to perform the MI task before participants re-entered the VE, which resembled the Embodied/Control scene but without the virtual mirror so participants could only focus on their virtual hands upon the table. Training consisted of 30 randomly presented trials, with 15 trials per class (left/right-hand grasp). Each trial included a 5-second rest period followed by a 5-second MI task period. Participants focused on a cross positioned between two virtual hands. When an arrow appeared pointing to one hand (visual cue), they were instructed to repeatedly imagine a grasping movement while observing at the same time the hand move (MI task; Figure 1E). After completing the trials, participants exited the VE and verbally responded to an embodiment question (“MIQ1—I felt like the body that I was seeing was my own body.") on a 7-point Likert scale.

##### 3.2.2.6 Online BCI

Participants re-entered the same virtual environment (VE) used during the MI training phase and repeated the task. In this phase, real-time feedback was provided by a machine learning classifier, trained on data collected during the MI training phase under the Embodied condition. This testing phase included two sessions, each offering distinct types of feedback based on the classifier's outputs (Bendor et al., 2025). As BCI performance results are not the focus of the present analysis, further details regarding the machine learning methods are not included here; these methods strictly followed those previously described by Vagaja et al. (2024).

#### 3.2.3 Experimental setup

##### 3.2.3.1 EEG equipment and acquisition

The EEG setup followed the same configuration as our pilot study, using 32 active electrodes arranged according to the 10–20 system, with the reference electrode over the left mastoid. EEG signals were recorded at a 250 Hz sampling rate using a LiveAmp 32 EEG wireless amplifier (Brain Products GmbH, Gilching, Germany) and BrainVision Recorder software (Brain Products GmbH, Gilching, Germany). Moreover, the online signal processing and classification was performed by NeuXus, a python based EEG signal processing tool (Legeay et al., 2022). To prevent interference with the VR headset, the electrodes were carefully positioned underneath it (Figure 1).

##### 3.2.3.2 VR scene and equipment

The experiment was conducted in the same VE as our Pilot study, where participants were seated in front of a virtual desk, facing a mirror resting on the table, within a room that replicated their real-world environment. This VE was developed using the Unity 3D game engine and is publicly available online^3^. Participants interacted with a gender-matched avatar generated via Ready Player Me2, with feedback provided through an Oculus Rift CV1 headset (Oculus VR, a subsidiary of Meta, Inc., United States), resorting to Oculus Touch controllers and Constellation sensors for hand tracking.

### 3.3 Embodiment and presence questionnaires

To evaluate SoE, 16 questions were taken from Peck and Gonzalez-Franco (2021), along with five additional questions adapted from the Multimodal Presence Scale (MPS) (Makransky et al., 2017). Participants provided verbal responses using a 7-point Likert scale.

From this questionnaire, seven features were computed:

- Appearance = (E1 + E2 + E3 + E4 + E5 + E6 + E9 + E16)/8
- Response = (E4 + E6 + E7 + E8 + E9 + E15)/6
- Ownership = (E5 + E10 + E11 + E12 + E13 + E14)/6
- Multi-sensory = (E3 + E12 + E13 + E14 + E15 + E16)/6
- Agency = (E3 + E13)/2
- Embodiment = (Appearance + response + ownership + multi-sensory)/4
- Physical Presence = (P1 + P2 + P3 + P4 + P5)/5

In addition, for the newly recorded data exclusively, one more feature was added, referring to the SoE felt by the subjects during the MI training phases (“MIQ1—I felt like the body that I was seeing was my own body."):

- Embodiment MI = MIQ1

### 3.4 EEG signals analysis

The analysis focused on the MI training phases to address the study's objectives. Specifically, the MI training conducted after the Embodied condition is referred to as MI Embodied, while the MI training following the Control condition is termed MI Control. Moreover, the dataset from Vagaja et al. (2024) also underwent the following signal pre-processing, ensuring all signals were processed using the same strategies when analyzing both Recorded and Combined Datasets.

EEG signals were processed using the EEGLAB toolbox (v2023.1) (Delorme and Makeig, 2004), in MATLAB version R2022a and R2023b.

#### 3.4.1 Pre-processing

**Figure 2:**
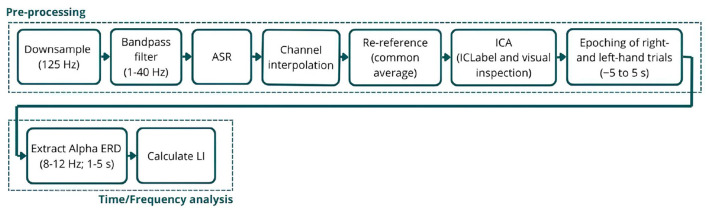
Schematic representation of the EEG signal processing pipeline. The **(top)** row illustrates the pre-processing steps applied to the raw EEG signals, and the **(bottom)** row outlines the time-frequency analysis used to analyze Alpha ERD.

The signals were first downsampled to 125 Hz, filtered between 1 and 40 Hz, and cleaned from noise and artifacts using the Artifact Subspace Reconstruction (ASR) algorithm (Chang et al., 2019). Using this technique, channels were removed if they remained flat for more than 5 s, contained artifacts in over 15% of windows, had a correlation below 0.5 with other channels, or exhibited excessive line noise. A burst criterion of 10 standard deviations was used to detect artifacts without applying high-pass filtering or segment removal, aiming to preserve the signal entirely. Next, eliminated channels were interpolated, and signals were re-referenced to the common average, followed by Independent Component Analysis (ICA) to further remove artifactual components. Components identified by ICLabel (Pion-Tonachini et al., 2019) as eye or muscle artifacts with a probability greater than 90% were flagged for automatic rejection. Additionally, all components were visually inspected, and up to a maximum of six components were manually selected for removal. Finally, the signals were epoched from −5 to 5 s for both the left- and right-hand trials, corresponding to the trial structure (5s baseline followed by 5s MI training) and ensuring the entire imagery period is captured. Each epoch was visually inspected, and bad epochs were removed (Figure 2).

#### 3.4.2 Time/frequency analysis

The Event-Related Spectral Perturbation (ERSP) was extracted from the pre-processed signals, isolating the Alpha band range (8–12 Hz), as it is the most responsive during MI tasks, and converting it to ERD using Equation 1. The ERD values represent the percentage decrease in Alpha power during the MI task relative to baseline (the symmetrical seconds before the MI period) (Pfurtscheller and da Silva, 1999). To further analyze it, the average ERD between 1 and 5 s for each electrode was calculated, enabling the creation of ERD scalp maps for each subject and trial. The first second following the MI task trigger was excluded, as participants require some time to initiate the task (reaction time), leading to an initial peak in band power unrelated to the ERD of interest.


(1) $$\begin{array}{l} ERD\left(\%\right)=\left(10^{ERSP/10}-1\right)*100 \end{array}$$

Additionally, the ERD power over C3 and C4 during the MI task was compared to the baseline (0% ERD), aiming to examine each participant's ability to generate significant ERD. These electrodes were chosen for analysis, similar to the Pilot study (Vagaja et al., 2024), as they are located over the left and right sensorimotor cortices, respectively, and are the most responsive during MI tasks.

Lastly, the Lateralization Index (LI) was calculated using Equation 2. LI is a common metric in neural imaging studies that quantifies the imbalance in neural activation between hemispheres (Doyle et al., 2005), where a positive LI value indicates greater contralateral desynchronization (Figure 2).


(2) $$\begin{array}{l} LI=\frac{ERD_{C3\left(left\right)}-ERD_{C4\left(left\right)}+RD_{C4\left(right\right)}-ERD_{C3\left(right\right)}}{2} \end{array}$$

### 3.5 Linear modeling

To further investigate the relationship between embodiment strength (measured through the questionnaire) and brain activity metrics (ERD and LI values), linear models were developed using the Combined Dataset. Two approaches were used, Simple Linear Regression (LR; Equation 3), serving as baseline, and Linear Mixed Effects (LME) models (Equation 4). LME models extend linear regression by incorporating random effects, allowing them to account for individual variations within population subgroups, which may arise due to the between-subject design of Vagaja et al. (2024)'s dataset. For ERD, models for each hand trail were applied separately to account for possible differences in the correlation between SoE and a specific hand, since hand dominance can influence ERD modulation during MI tasks (Schomer et al., 2017; Stancák and Pfurtscheller, 1996; Crotti et al., 2022; Stancak and Pfurtscheller, 1996; Bai et al., 2005).


(3) $$\begin{array}{r} ERD_{left}\left(\%\right)=\beta_{0}+\beta_{1}*Embodiment\\ ERD_{right}\left(\%\right)=\beta_{0}+\beta_{1}*Embodiment\\ LI\left(\%\right)=\beta_{0}+\beta_{1}*Embodiment \end{array}$$

(4) $$\begin{array}{r} ERD_{left}\left(\%\right)=\beta_{0}+\beta_{1}*Embodiment+\left(1|Subject\right)\\ ERD_{right}\left(\%\right)=\beta_{0}+\beta_{1}*Embodiment+\left(1|Subject\right)\\ LI\left(\%\right)=\beta_{0}+\beta_{1}*Embodiment+\left(1|Subject\right) \end{array}$$

### 3.6 EEG feature discriminability

Similarly to the Pilot Study, the EEG classification accuracy during MI training was computed for the Extended Dataset to distinguish between left- and right-hand MI trials. This analysis aimed to assess the discriminability of MI-related EEG features within each condition (MI Control and MI Embodied). EEG data for each subject were band-pass filtered between 8 and 28 Hz to target activity within the Alpha and Beta bands, followed by feature extraction using Common Spatial Patterns (CSP), retaining six spatial filters. CSP is a commonly used algorithm for standard MI-BCI feature extraction, as it discriminates movement-related spatial patterns and maximizes the difference between two classes (Ramoser et al., 2000). Next, a Shrinkage Linear Discriminant Analysis (LDA) classifier was trained on the extracted CSP features, and its performance was evaluated using Monte Carlo cross-validation with 10 iterations and a test set comprising 20% of the data in each fold. For each subject, the final classification accuracy was calculated as the mean accuracy in all folds.

### 3.7 Statistical analysis

To determine the appropriate statistical methods for comparing conditions (MI Control vs. MI Embodied), the normality and homoscedasticity of ERD and LI values were assessed using the Kolmogorov–Smirnov and Levene tests, respectively. These tests were conducted separately on both the Extended Dataset and the Pilot Study dataset to investigate if feature distributions followed normality and had consistent variance across conditions. Although some features met the criteria for normality and homoscedasticity, the results were inconsistent within and between datasets. Additionally, the small sample size in each dataset (only 13 subjects per condition) led to the choice of non-parametric tests for all comparisons to ensure methodological consistency. As a result, all comparisons between conditions and feature analyses were performed using the Mann–Whitney *U*-test. To evaluate participants' ability to induce ERD during the MI task, a Single Sample Wilcoxon Signed-Rank test was applied. Finally, the linear models were evaluated using AIC, BIC, and *R*^2^, which provide insights into the models' fit to the data. The fitted models also generated *p*-values for each predictor, indicating their statistical significance in predicting the response variables (ERD and LI values). For all tests and comparisons, it was used a significance level of 0.05 (*p*-value < 0.05).

## 4 Results

The results are presented in four sections. First, we report the subjective responses from the embodiment questionnaire to confirm the successful induction of the embodiment illusion. Second, we examine the impact of prior embodiment on EEG activity, focusing on ERD and its lateralization. Third, we present the outcomes of the LME modeling to explore relationships between embodiment strength and EEG metrics. Finally, we report the performance of the trained classifier in distinguishing EEG patterns between left- and right-hand MI classes during training.

### 4.1 Sense of embodiment induction and its validation

The subjective responses through the questionnaire confirmed successful induction of the embodiment illusion in the Extended Dataset, with significantly higher scores observed in the Embodied condition compared to the Control condition across multiple embodiment dimensions: appearance (Control: 3.30 ± 1.35, Embodied: 4.40 ± 1.15; *U* = 175.00, *p* = 0.02), response (Control: 3.22 ± 1.54, Embodied: 5.08 ± 0.95; *U* = 156.50, *p* < 0.001), ownership (Control: 3.23 ± 1.67, Embodied: 5.34 ± 0.82; *U* = 153.50, *p* < 0.001), multi-sensory integration (Control: 3.27 ± 1.48, Embodied: 5.50 ± 0.91; *U* = 149.50, *p* < 0.001), agency (Control: 3.13 ± 1.55, Embodied: 4.77 ± 1.29; *U* = 166.00, *p* = 0.01), and overall embodiment (Control: 3.25 ± 1.44, Embodied: 5.08 ± 0.88; *U* = 151.00, *p* < 0.001). However, presence scores remained high without significant differences between conditions (Control: 4.56 ± 1.46, Embodied: 5.33 ± 1.20; *U* = 194.50, *p* = 0.12). Similarly, SoE scores during the MI training were elevated and comparable in both MI Control (4.80 ± 1.61) and MI Embodied (4.73 ± 1.67; *U* = 235.00, *p* = 0.93) conditions.

**Figure 3:**
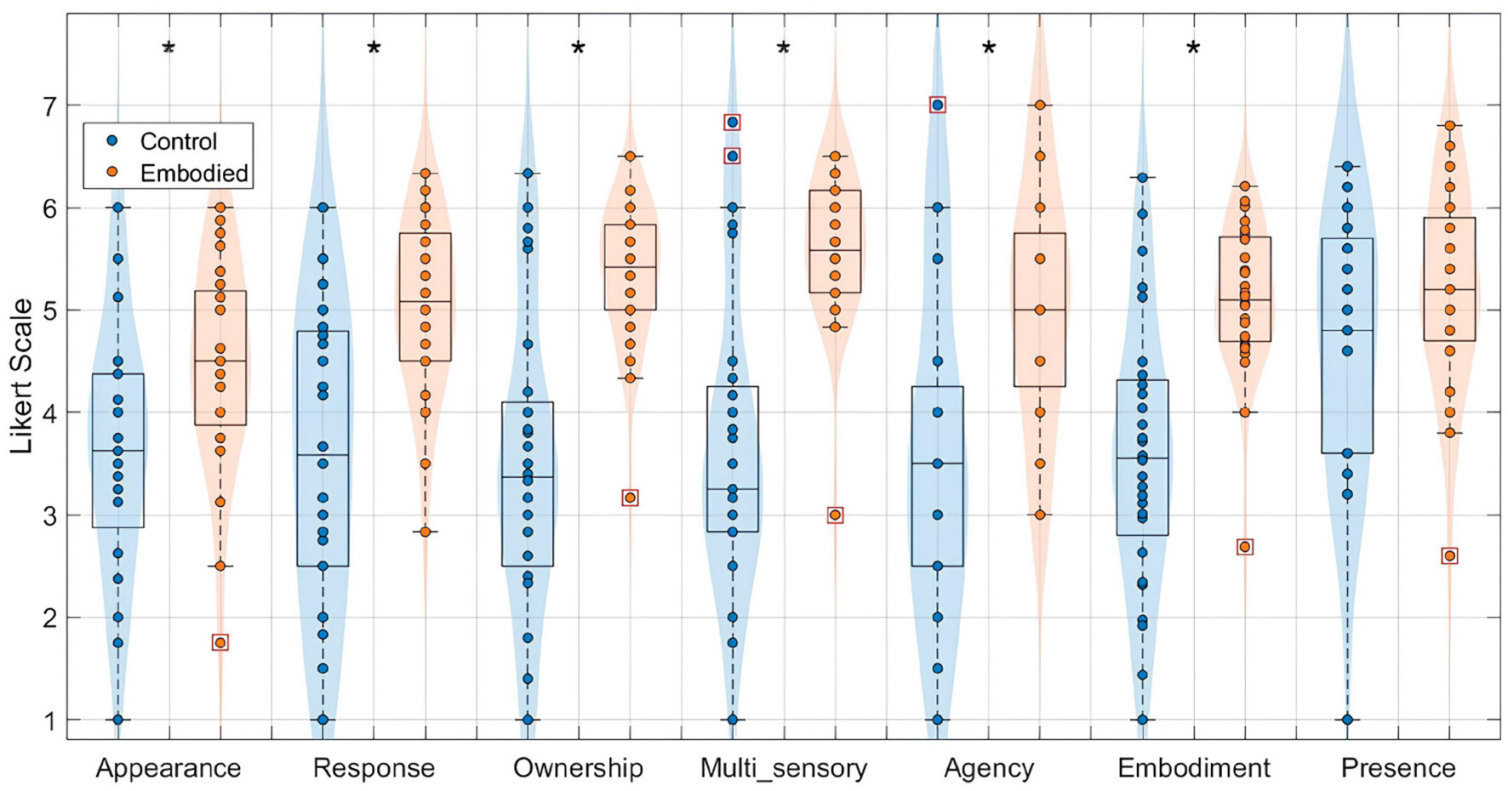
Distribution of features extracted from the embodiment questionnaire for the Combined Dataset, specifically appearance, response, ownership, multi-sensory integration, agency, embodiment, and presence, in a 7-point Likert Scale. Values for the Control condition are represented in blue, while the Embodied condition is in orange. Features with statistically significant differences between conditions, as determined by the Mann–Whitney *U*-test, are marked with an asterisk (*) (*p*-value < 0.05). Each box plot shows the median, interquartile range, and outliers (denoted by red boxes).

Given that the Extended Dataset demonstrated effective manipulation of the embodiment illusion, consistent with the Pilot study (Vagaja et al., 2024), it was appropriate to combine both datasets into the “Combined Dataset" for a consolidated analysis. This integrated analysis further reinforced the differences in SoE between conditions, as illustrated in Figure 3. Specifically, the Embodied condition yielded significantly higher scores for appearance (Control: 3.67 ± 1.22, Embodied: 4.46 ± 1.01; *U* = 634.00, *p* = 0.01), response (Control: 3.56 ± 1.42, Embodied: 5.02 ± 0.84; *U* = 565.50, *p* < 0.001), ownership (Control: 3.48 ± 1.45, Embodied: 5.36 ± 0.70; *U* = 523.00, *p* < 0.001), multi-sensory integration (Control: 3.64 ± 1.45, Embodied: 5.60 ± 0.72; *U* = 526.00, *p* < 0.001), agency (Control: 3.57 ± 1.62, Embodied: 5.05 ± 1.17; *U* = 591.50, *p* < 0.001), and overall embodiment (Control: 3.59 ± 1.31, Embodied: 5.11 ± 0.73; *U* = 531.00, *p* < 0.001). Only the presence scores did not significantly differ and remained high in both conditions (Control: 4.64 ± 1.26, Embodied: 5.18 ± 0.97; *U* = 703.00, *p* = 0.12).

### 4.2 Effect of prior embodiment on ERD

In general, subjects in the Extended Dataset exhibited clear ERD induction with the expected ERD and LI patterns; however, prior embodiment did not result in significant differences in ERD within the motor-related C3 and C4 channels during the MI tasks. A similar scenario was observed in the Combined Dataset, with no evidence of successful SoE induction before MI training. Nonetheless, a slight trend toward greater LI variability was noted in the MI Embodied condition, suggesting more heterogeneous responses to embodiment priming. While no group-level effect was found, individual differences in embodiment susceptibility may have influenced the neural responses, providing a relevant lead for future research.

Starting by presenting the ability to induce ERD in the Extended Dataset, seven out of the 13 subjects successfully generated significant ERD in both C3 and C4 electrodes during all hand trials and conditions (right- and left-hand trials in both MI Control and MI Embodied conditions), demonstrating their ability to induce ERD correctly.

Subjects 02, 03, and 10 lacked correct ipsilateral ERD. Subject 02 failed to produce significant ipsilateral ERD in the MI Embodied condition (C3 in left trials and C4 in right trials), and also lacked significant ERD in the ipsilateral region (C4) during right-hand trials in the MI Control condition (*p*-value = 0.34). Subject 03 did not exhibit ipsilateral ERD (C3) during left-hand trials in either condition, while Subject 10 failed to achieve its significance during the MI Control condition (*p*-value = 0.59). Additionally, Subjects 16, 17, and 19 represent more concerning cases, with imperfect contralateral ERD. Subject 16 showed no contralateral ERD (C3) during right-hand trials in the MI Control condition, along with non-significant ERD in the ipsilateral region for left trials (C3) in MI Control (*p*-value = 0.31) and for right trials (C4) in MI Embodied (*p*-value = 0.07). Subject 17 lacked contralateral ERD (C4) during left-hand trials in MI Control and ipsilateral ERD (C4) during right-hand trials in MI Embodied, with additional non-significant ERD in C3 for left-hand trials in MI Embodied (*p*-value = 0.18). Similarly, Subject 19 failed to generate significant contralateral ERD (C4) for left-hand trials in MI Control (*p*-value = 0.93) and ipsilateral ERD (C4) for right-hand trials in MI Embodied (*p*-value = 0.15), also lacking ERD in C3 for left-hand trials in MI Control.

**Figure 4:**
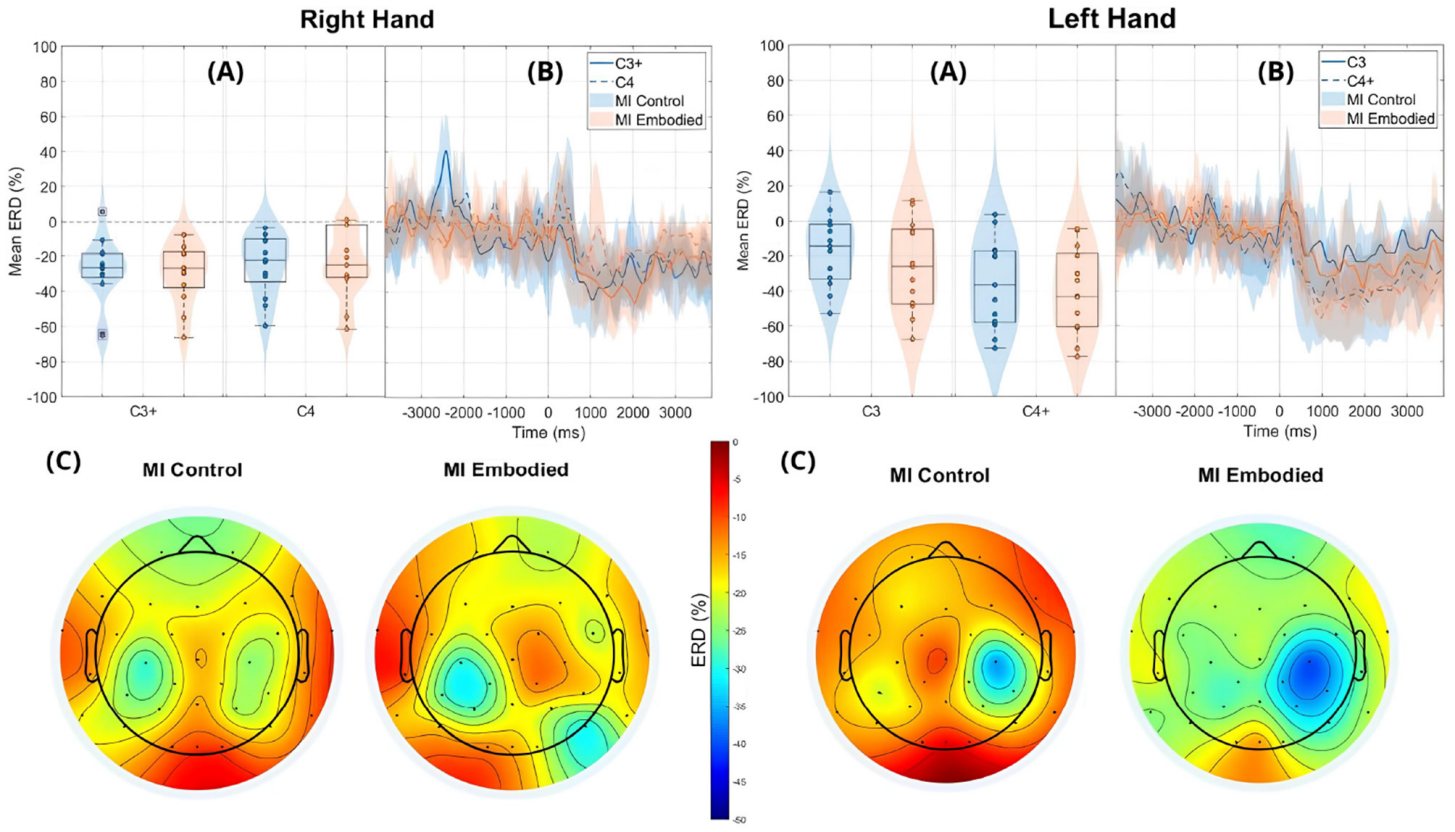
ERD grand averages across MI Control (blue) and MI Embodied (orange) conditions during right- and left-hand MI trials for the Extended Dataset. “+" indicates contralateral electrode. **(A)** Distribution of ERD values for MI Control and MI Embodied conditions at C3 and C4 electrodes from 1,000 ms to 5,000 ms during MI trials. The horizontal line represents the baseline (0% ERD), and red boxes denote outliers. **(B)** Time course of ERD during MI trials (from 0 ms to 4,000 ms). Lines represent mean ERD values within the Alpha band across all subjects, with shaded regions indicating the 25th and 75th percentiles. Vertical dashed lines mark the stimulus onset (0 ms), and the horizontal line indicates baseline ERD during the resting state (0% ERD). **(C)** Topographic distribution of mean ERD. The color scale ranges from 0% (red, no ERD) to –50% (blue, indicating strong ERD).

**Figure 5:**
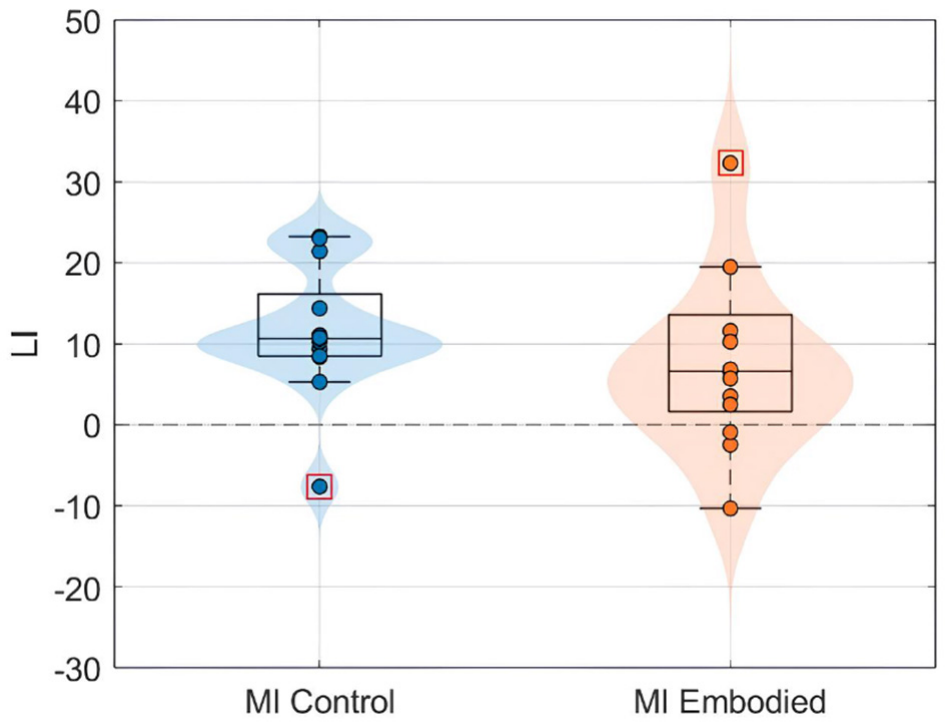
LI value (%) distributions for the MI Control (blue) and MI Embodied (orange) conditions in the Extended Dataset. The horizontal dashed line represents no ERD lateralization, and red boxes denote outliers.

Still, subjects overall produced significant ERD, as shown in Figure 4 and Table 1, which summarize ERD analysis for right- and left-hand trials. The C3 and C4 ERD distribution in Figure 4A shows no substantial differences between MI Embodied and MI Control conditions, except for slightly stronger ERD in MI Embodied during left-hand trials (−40.00%; Table 1) and broader ERD distribution in left-hand trials compared to right-hand ones. Figure 4B illustrates the expected ERD temporal pattern, with Alpha power suppression beginning shortly after the trigger (0 ms) and remaining suppressed throughout the trial. Nevertheless, it presents rapid fluctuations, continually returning to baseline at a rapid rate. Scalp maps in Figure 4C confirm ERD induction over sensorimotor areas, though in MI Embodied during right-hand trials, the strongest desynchronization shifts toward the parieto-occipital region rather than directly over the ipsilateral electrode (C4). Notably, the contralateral electrode exhibited stronger ERD than the ipsilateral one across both hand trials and conditions. This ERD lateralization is further supported by positive LI values (Figure 5 and Table 1), though lateralization was slightly lower in MI Embodied (LI = 14.53%) than MI Control (LI = 11.43%). Moreover, Figure 5 indicates greater LI variability when the prior embodiment is present (MI Embodied), suggesting more dispersed lateralization effects. Despite these trends, no significant differences between conditions were observed for either hand trial.

**Table 1:** Mean ERD (%) and LI (%) values, *U*-statistics, and *p*-values from the Mann–Whitney *U*-test comparing MI Control and MI Embodied conditions in the Extended Dataset.

	**Mean (%)**		**Comparison**	
	**MI control**	**MI embodied**	*U* **-statistics**	*p* **-value**
C3 L	–17.04	–26.04	192.00	0.41
C4 L	–35.70	–40.00	184.00	0.68
C3 R	–28.70	–29.34	177.00	0.96
C4 R	–24.51	–14.25	173.00	0.92
LI	11.43	14.53	199.00	0.24

ERD is reported for contralateral electrodes [C3 for right-hand trials (C3 R) and C4 for left-hand trials (C4 L)] and ipsilateral electrodes [C4 for right-hand trials (C4 R) and C3 for left-hand trials (C3 L)].

**Figure 6:**
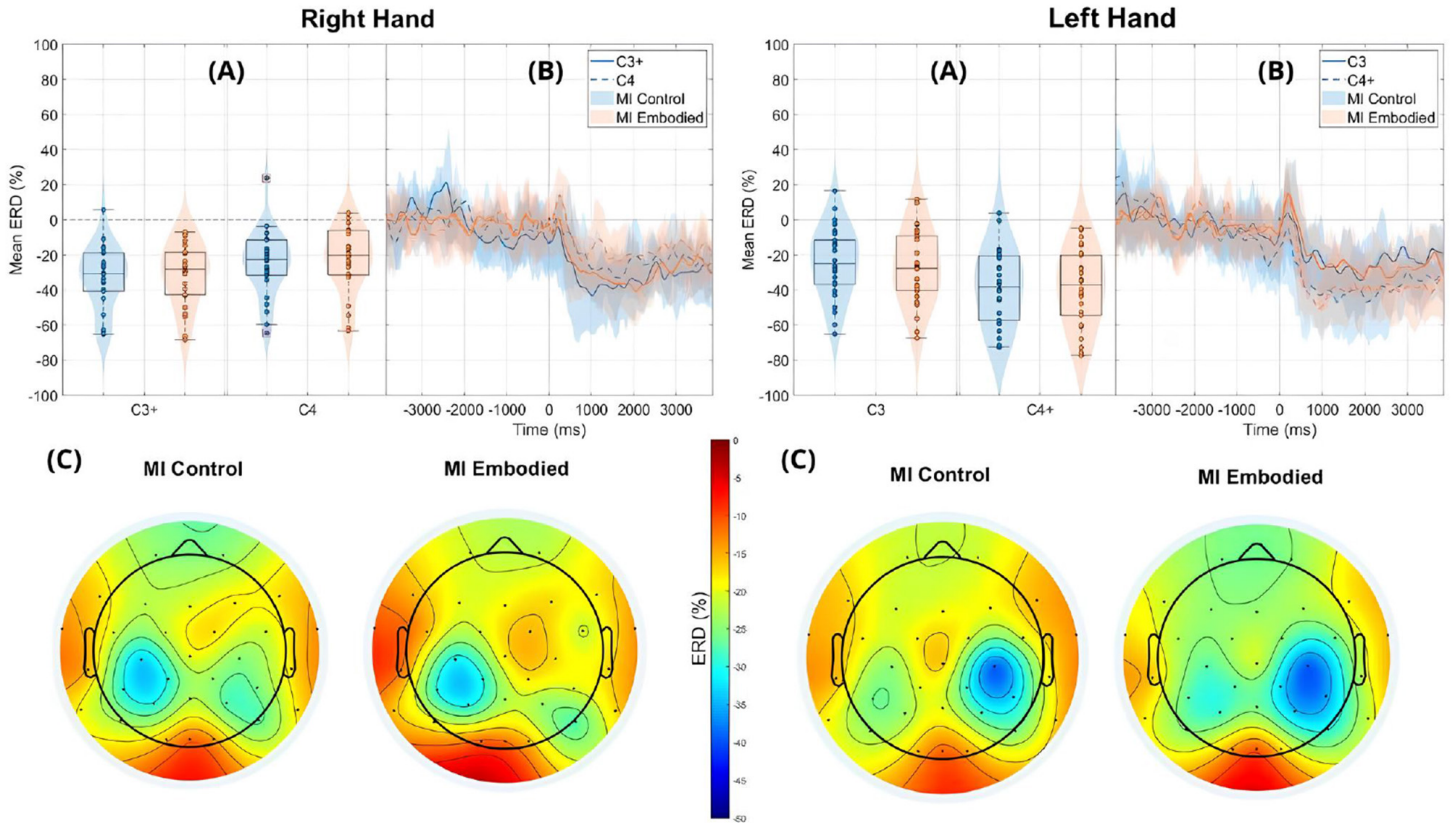
ERD grand averages across MI Control (blue) and MI Embodied (orange) conditions during right- and left-hand MI trials for the Combined Dataset. “+” indicates contralateral electrode. **(A)** Distribution of ERD values for MI Control and MI Embodied conditions at C3 and C4 electrodes from 1,000 ms to 5,000 ms during MI trials. The horizontal line represents the baseline (0% ERD), and red boxes denote outliers. **(B)** Time course of ERD during MI trials (from 0 ms to 4,000 ms). Lines represent mean ERD values within the Alpha band across all subjects, with shaded regions indicating the 25th and 75th percentiles. Vertical dashed lines mark the stimulus onset (0 ms), and the horizontal line indicates baseline ERD during the resting state (0% ERD). **(C)** Topographic distribution of mean ERD. The color scale ranges from 0% (red, no ERD) to –50% (blue, indicating strong ERD).

**Figure 7:**
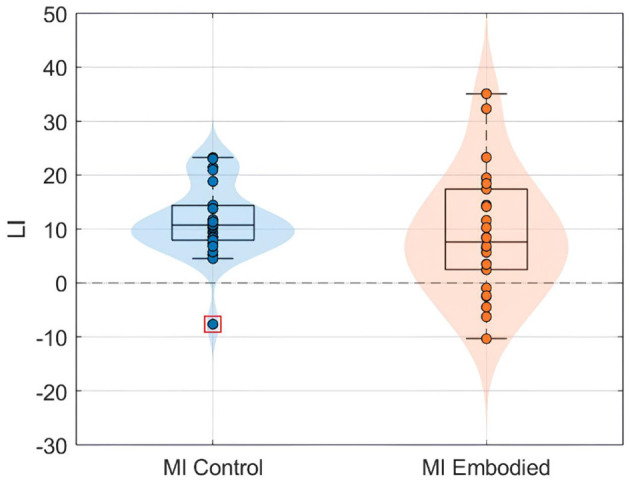
LI value (%) distributions for the MI control (blue) and MI embodied (orange) conditions in the Combined Dataset. The horizontal dashed line represents no ERD lateralization, and red boxes denote outliers.

The ERD analysis of the Combined Dataset (Figure 6 and Table 2) also confirms correct ERD induction for both hand trials and conditions, demonstrating that merging the datasets does not affect ERD induction. Figure 6 illustrates the expected MI task morphology, with strong ERD occurring shortly after the provided cue (0 ms) over sensorimotor areas. However, during right-hand trials, power reduction shifts toward the parieto-occipital region, limiting overlap with the C4 electrode, similar to patterns observed in the Extended Dataset (Figure 4). During left-hand trials, the contralateral electrode (C4) exhibited the strongest ERD, with MI Control showing slightly weaker ERD than MI Embodied (MI Control: −38.67%; MI Embodied: −39.01%, Table 2). Conversely, MI Control showed stronger ERD in right-hand trials, though the differences were minor (Table 2). Furthermore, left-hand trials demonstrated greater ERD dispersion compared to right-hand. As expected, contralateral electrodes exhibited stronger desynchronization than ipsilateral ones across both hand trials and conditions, confirming ERD lateralization. This is further supported by positive LI values (Figure 7 and Table 2). While MI Embodied showed a slightly higher LI (12.54%) than MI Control (11.22%; Table 2), the values remain close. The main difference between conditions was the broader LI dispersion in MI Embodied (Figure 7), mirroring trends in the Extended Dataset (Figure 5). However, no statistically significant differences were found between conditions, as confirmed by the Mann–Whitney *U*-test (Table 2).

**Table 2:** Mean ERD (%) and LI (%) values, *U*-statistics, and *p*-values from the Mann–Whitney *U*-test comparing MI Control and MI Embodied conditions in the Combined Dataset.

	**Mean (%)**		**Comparison**	
	**MI control**	**MI embodied**	*U* **-statistics**	*p* **-value**
C3 L	–24.31	–27.29	714.00	0.65
C4 L	–38.67	–39.01	689.00	1.00
C3 R	–33.00	–30.81	661.00	0.62
C4 R	–24.93	–17.48	645.00	0.43
LI	11.22	12.53	748.00	0.28

ERD is reported for contralateral electrodes [C3 for right-hand trials (C3 R) and C4 for left-hand trials (C4 L)] and ipsilateral electrodes [C4 for right-hand trials (C4 R) and C3 for left-hand trials (C3 L)].

### 4.3 Linear relationship between embodiment strength with ERD and LI

**Figure 8:**
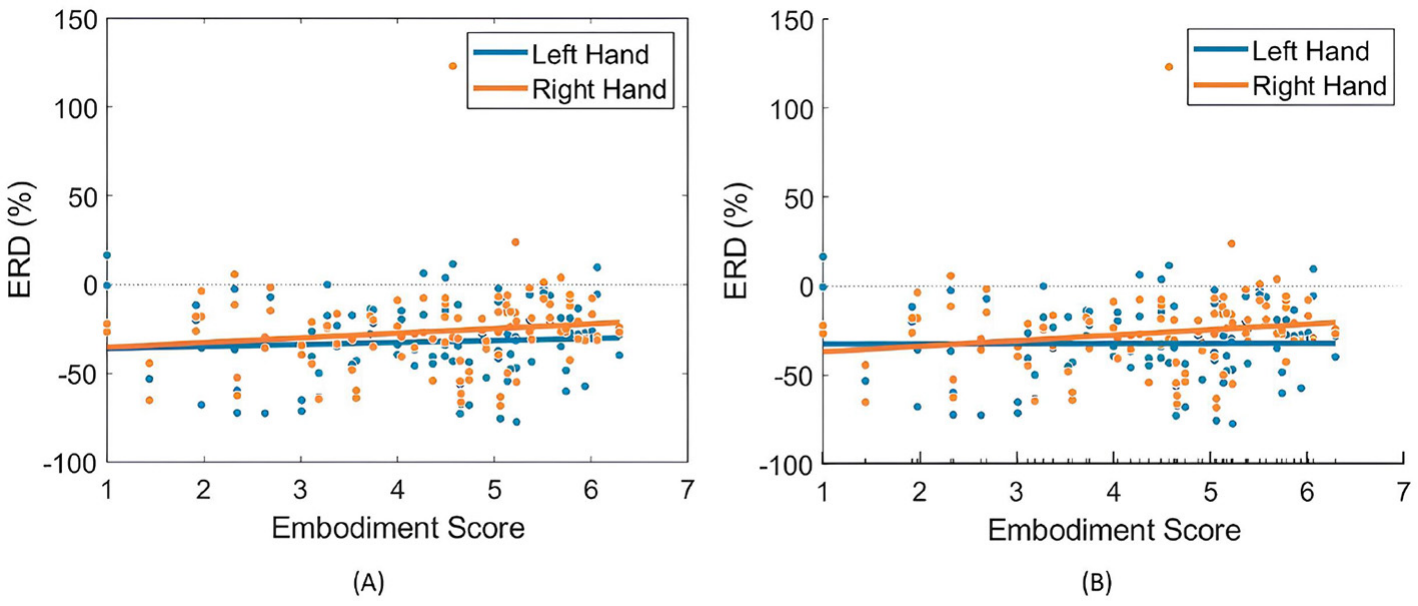
Relationships between embodiment score and ERD (%) values across both right- and left-hand trials. **(A)** Presents the fitted LR model (Equation 3), while **(B)** illustrates the partial dependence of embodiment score based on the results of the LME model (Equation 4).

When analyzing the relationship between embodiment strength and ERD in the Combined Dataset, both LR and LME models exhibited near-flat trendlines, with slopes approaching zero, indicating a negligible correlation (Table 3). Still, models showed a weak, non-significant positive correlation between embodiment and ERD, particularly in right-hand trials using LME models (Table 3; Figure 8). This slightly stronger correlation in the right-hand trials highlights a potential variability in hand response to MI tasks and prior SoE.

**Table 3:** Linear models (LR and LME) evaluating the relationship between embodiment and ERD/LI.

**Outcome (model)**	**Model variables**					**Model evaluation**		
	**Variable**	**Estimate**	**SE**	*t* **-Statistics**	*p* **-value**	**AIC**	**BIC**	*R* ^2^
ERD_*left*_ (LR)	β_0_	–37.22	7.53	–4.94	0.00*	939.66	944.95	0.01
	Embodiment	1.13	1.66	0.68	0.50			
ERD_*left*_ (LME)	β_0_	–33.28	7.51	–4.43	0.00*	911.85	922.43	0.59
	Embodiment	0.06	1.57	0.04	0.97			
ERD_*right*_ (LR)	β_0_	–38.05	8.04	–4.73	0.00*	953.24	958.53	0.02
	Embodiment	2.65	1.78	1.49	0.14			
ERD_*right*_ (LME)	β_0_	–40.67	8.46	–4.81	0.00*	942.59	953.16	0.35
	Embodiment	3.10	1.82	1.71	0.09			
LI (LR)	β_0_	13.23	7.77	1.70	0.10	438.01	441.92	0.00
	Embodiment	–0.31	1.72	–0.18	0.86			
LI (LME)	β_0_	11.14	7.60	1.47	0.15	441.20	449.01	0.06
	Embodiment	0.15	1.66	0.09	0.93			

^*^Indicates significant differences at *p* < 0.05.

When comparing LR and LME models, both exhibited similar correlations between ERD and embodiment, yet LME models demonstrated a better fit (Table 3) and a more pronounced relationship between variables. This underscores the advantage of LME models in accounting for individual differences in embodiment responses.

**Figure 9:**
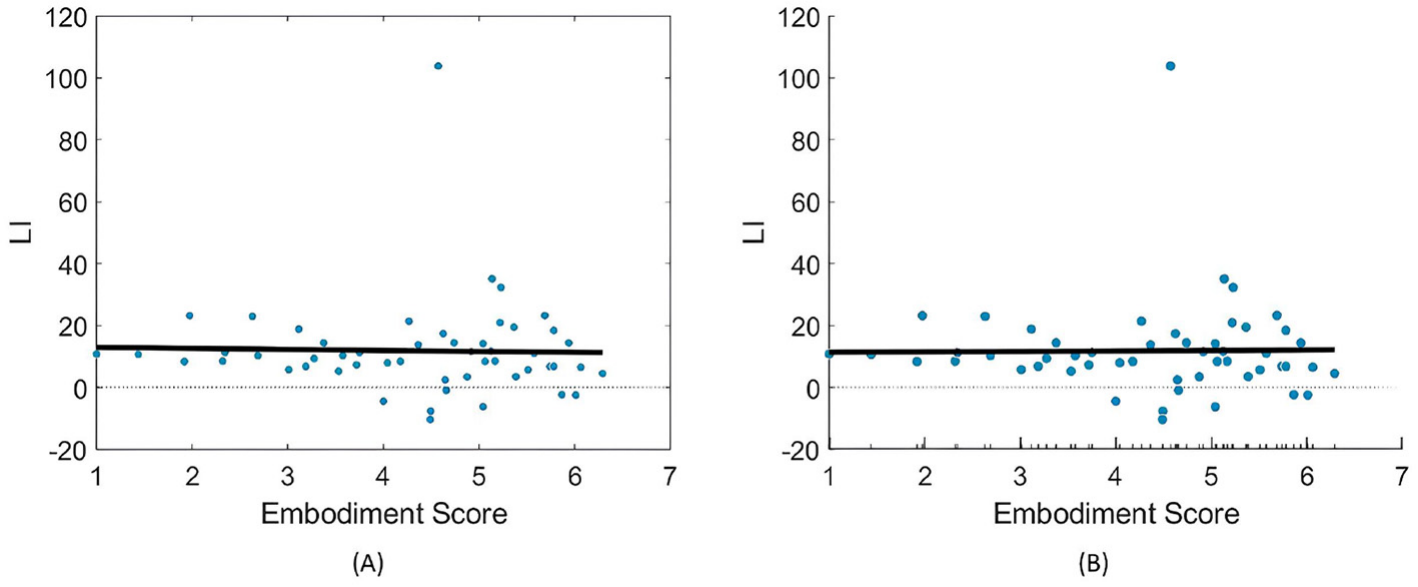
Relationships between embodiment score and LI (%) values across both right- and left-hand trials. **(A)** Presents the fitted LR model (Equation 3), while **(B)** illustrates the partial dependence of embodiment score based on the results of the LME model (Equation 4).

For LI, neither model found a significant correlation with embodiment strength (Figure 9 and Table 3). Interestingly, the models showed opposing trends in LI response to SoE, with LME indicating a negative correlation (−0.31) and LR suggesting a positive correlation (0.15), further complicating any supposition.

### 4.4 Effect of prior embodiment on EEG feature discriminability

**Figure 10:**
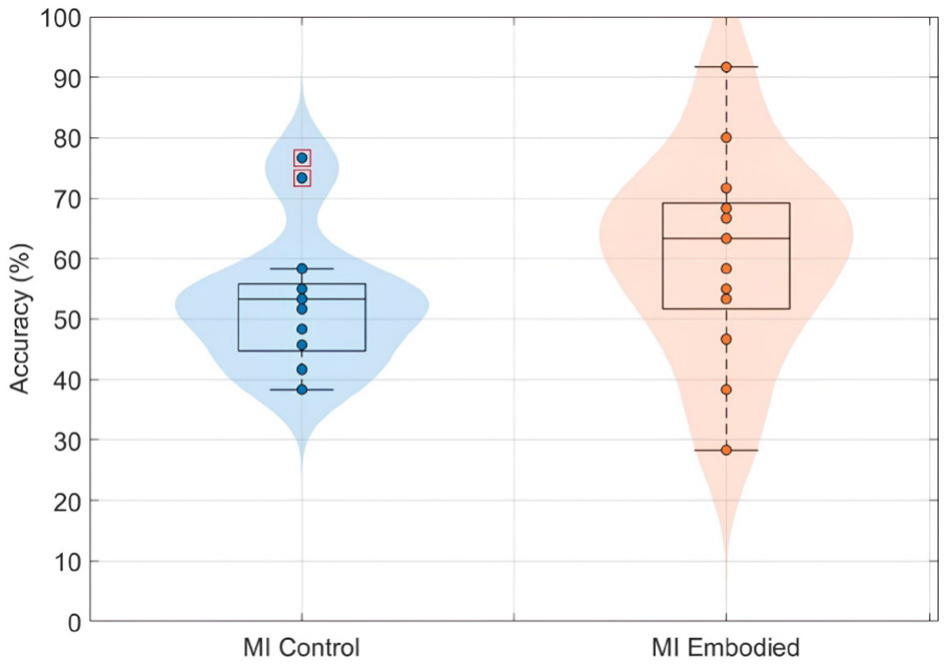
BCI classification accuracies (%) distribution using shrinkage LDA for the MI Control (blue) and MI Embodied (orange) conditions in the Extended Dataset. Each point represents a participant's mean accuracy across 10 Monte Carlo cross-validation folds. Red boxes indicate outliers.

Similar to the findings for ERD and LI, prior embodiment induction did not affect the discriminability of EEG features for BCI classification. Both conditions showed similar low, non-differing classification accuracy. Figure 10 shows the distribution of EEG classification accuracies obtained using the shrinkage LDA classifier for both conditions in the Extended Dataset. While the MI Embodied condition (60.64 ± 16.89%) showed a slightly higher average accuracy compared to the MI Control condition (53.13 ± 11.38%), which could suggest a positive influence of prior embodiment induction, this difference was not statistically significant (*U*-statistics = 147.00; *p*-value = 0.15). However, overall classification accuracy was very low, probably reflecting the general difficulty that participants had in producing discriminable ERD patterns. In particular, the MI Embodied condition presented a greater distribution of accuracies between subjects, also mirroring the variability observed in the LI values.

## 5 Discussion

The SoE illusion was successfully induced in the Embodied condition and effectively disrupted in the Control condition, confirming the validity of the experimental manipulation. High presence scores in both conditions for the Recorded and Combined Datasets indicated strong immersion in the VE, further supporting the correct induction of the illusion, as presence and SoE share common induction factors such as head-tracking, depth perception, and sensory synchrony (Halbig and Latoschik, 2024; Pritchard et al., 2016). Since the illusion was well-established, and merging datasets did not affect its induction, EEG metrics from MI Embodied and MI Control conditions can be linked to SoE.

However, results showed no significant ERD differences between conditions, suggesting prior embodiment did not influence ERD induction during MI training. Although the Extended Dataset exhibited stronger ERD in the MI Embodied condition for left-hand trials, this difference was not statistically significant. A similar trend in the Combined Dataset hints at a potential subtle effect of prior SoE on MI performance. Additionally, LI analysis showed slightly stronger lateralization in the MI Embodied condition in both datasets, though differences were too small to be conclusive. These findings align with Vagaja et al. (2024), ruling out confounding effects from a between-subject design and statistical power limitations. Therefore, the results indicate that incorporating a prior embodiment induction phase when design and VR-based MI-BCI training procedure may not be necessary, as embodiment during MI training alone appears sufficient to elicit ERD.

Interestingly, greater LI distribution was observed when prior embodiment was present for both datasets (MI Embodied; Figures 5, 7), further supported by Vagaja et al. (2024). This suggests embodiment may introduce individual variability in response to VR feedback during MI training. Previous research indicates that embodied VR feedback strengthens Alpha ERD lateralization, particularly when combined with vibrotactile feedback (Vourvopoulos et al., 2022; Batista et al., 2024), while MI training without feedback does not (Stefano Filho et al., 2020). Continued MI training with embodied VR feedback also improves ERD lateralization (Meng and He, 2019), with factors like task structure, feedback type, MI duration, and handedness also playing a role (Nam et al., 2011; Stancak and Pfurtscheller, 1996). These factors likely contribute to the greater LI variability observed in the MI Embodied condition. This variability highlights the individualized effects of embodied VR feedback, which is introduced during the Embodied/Control phases and carried through to subsequent MI training phases. While embodied VR feedback enhances MI by creating a more immersive and relatable experience compared to abstract feedback, its effectiveness varies among participants. This underscores the importance of tailoring VR-based BCI training based on participant-specific characteristics to optimize outcomes. Future VR-BCI systems could incorporate pre-assessments of susceptibility to body illusions to identify users most likely to benefit from prior embodiment, enabling more personalized training protocols. Potential strategies might include standardized questionnaires (such as MI vividness), behavioral tasks, or neurophysiological measures [e.g., resting frontal alpha power (Hsu et al., 2022)]. However, further research is needed to establish reliable methods for predicting embodiment responsiveness, and effective techniques for measuring embodiment neurophysiologically are still lacking.

Linear models further confirmed no significant effect of prior SoE on ERD induction, showing no correlation between SoE strength and ERD values for either hand. Similarly, no significant correlation was found between embodiment strength and LI values, with near-flat trendlines. However, for right-hand ERD, a small positive correlation with embodiment strength was observed, suggesting a potential hand-specific response to SoE. Literature indicates right-handed individuals show greater lateralization for their dominant hand (Schomer et al., 2017; Stancák and Pfurtscheller, 1996; Crotti et al., 2022; Stancak and Pfurtscheller, 1996; Bai et al., 2005). Nonetheless, ERD analysis did not confirm this, as the Extended Dataset displayed greater lateralization for left-hand trials in MI Control and minimal differences in MI Embodied. The strongest ERD appeared at C4 during left-hand trials in both conditions, suggesting better MI performance with the non-dominant hand, complicating the interpretation of hand dominance in MI training. These inconsistencies may reflect individual variability or limited MI training, as untrained individuals often exhibit similar activation for both hands during MI tasks (Keskin et al., 2017). Additionally, hand dominance effects on ERD strength may vary depending on feedback and stimulation techniques (Grigorev et al., 2021), highlighting the complex relationship between hand dominance and ERD induction.

While results suggest individual variability in prior SoE's effect on ERD lateralization, they indicate that it does not significantly impact MI training, with embodied feedback alone being sufficient. However, poor model fit (high AIC/BIC, low *R*^2^) warrants cautious interpretation. Another key factor is the participants' limited ability to induce ERD during MI training in the Extended Dataset likely impacted results. This is likely due to their lack of prior MI experience, as research suggests continuous training is needed to enhance ERD modulation (Mokienko et al., 2013; Jochumsen et al., 2017). The combination of limited MI training and lower MI vividness may explain the weaker ERD observed, potentially masking any effect of prior embodiment. Furthermore, individual differences in MI vividness influence ERD induction, contributing to variability across participants (Rimbert and Lotte, 2022).

In addition, all participants in the Extended Dataset reported feeling embodied in both MI Control and MI Embodied conditions. Although a single general question may not be the most reliable SoE assessment, this suggests both conditions successfully induced SoE. Participants received congruent visuoproprioceptive feedback, known to be sufficient for SoE induction (Falcone et al., 2022; Carey et al., 2019), and observed the imagined hand moving, potentially creating a false visuomotor cue that reinforced the illusion. SoE is believed to develop rapidly due to fast multisensory integration. Studies indicate the RHI emerges within 19–23 s (Kalckert and Ehrsson, 2017; Finotti et al., 2023), while virtual embodiment in first-person perspectives can occur even faster, within 5 s (Keenaghan et al., 2020). Full-body illusions from visual-tactile stimuli, such as in this study, take around 25 s to develop–slower than body-part illusions (Kondo and Sugimoto, 2022). While short exposures can induce SoE, prolonged exposure strengthens it, particularly in static illusions like MI training (Perepelkina et al., 2018; Kalckert and Ehrsson, 2017; Finotti et al., 2023), with continuous sensorimotor feedback essential for maintaining embodiment (Eck and Pfister, 2024). Thus, SoE may have emerged within the first seconds of MI training in both conditions and likely strengthened over time, minimizing differences between them, which may help explain the lack of significant effects found.

Another consideration is that ipsilateral ERD during right-hand trials does not fully overlap with the C4 electrode, as it is more localized in posterior brain regions (parietal lobe; Figures 5, 7). This suggests C4 may not entirely capture ERD's spatial distribution, raising concerns about relying on a single electrode. This study used C3 and C4 electrodes for ERD calculation, following MI-BCI research standards (Yeom and Sim, 2008). Still, optimal ERD detection sites may not always align with C3/C4, especially given subject-specific spectral and spatial variations (Stefano Filho et al., 2020). This is particularly relevant for untrained participants, who often exhibit broader associative zone activation during MI tasks (Mokienko et al., 2013). Additional electrodes could provide a more comprehensive assessment of prior embodiment's influence on MI performance.

Lastly, and in line with the low ERD magnitudes observed, EEG feature classification performance was also poor, remaining only slightly above chance level in both conditions. While some participants achieved relatively high accuracies, others showed very low performance, highlighting substantial inter-subject variability. This variability was especially pronounced in the MI Embodied condition, resembling the distribution observed in the LI values and again suggesting that SoE integration is highly individual, with participants responding differently to the VR feedback during MI training. Although the MI Embodied condition showed slightly higher average accuracy compared to MI Control, potentially indicating a small benefit from prior embodiment, this difference was not statistically significant. These results mirror the patterns observed in both ERD and LI metrics, further supporting the conclusion that prior embodiment had minimal influence on MI-related cortical patterns, consistent with findings from the Pilot study (Vagaja et al., 2024).

In practical terms, the findings indicate that VR-BCI systems can rely on embodiment induced during MI training itself, without needing a separate prior embodiment induction phase. When design VR-based MI-BCI procedures, the focus should rely on providing congruent multisensory feedback during MI tasks to optimize ERD induction rather than adding separate embodiment protocols, potentially saving training time and simplifying system implementation. However, given the observed inter-subject variability, future systems may benefit from assessing users' MI vividness or susceptibility to body illusions beforehand, allowing the training protocol to be tailored for maximum effectiveness.

## 6 Conclusion

This study investigated the role of virtual embodiment as a priming mechanism prior to MI-BCI training in VR, with ERD serving as the primary neurophysiological marker. While SoE was successfully induced in the Embodied condition, the results did not reveal significant differences in ERD between conditions, suggesting that embodiment during MI-VR training is sufficient to elicit robust ERD, consistent with prior studies, and that additional embodiment priming beforehand does not substantially influence MI-related brain activity.

Interestingly, ERD lateralization and EEG classification performance exhibited greater variability in the Embodied condition, pointing to individual differences in the way users integrate embodied feedback. This suggests that while SoE may not have a uniform effect on MI-induced ERD, it could introduce nuanced variations in lateralization and brain patterns, which warrant further investigation. These results align with previous findings and reinforce the need for future research to explore personalized approaches in VR-BCI training.

Several limitations should be considered when interpreting these findings. The relatively small sample size, despite being larger than previous studies, may have limited the statistical power to detect subtle effects of prior embodiment. Additionally, the reliance on C3 and C4 electrodes for ERD measurement may not have fully captured individual differences in MI-related activation patterns. Future studies should explore more extensive electrode coverage and incorporate additional neurophysiological measures to assess the broader impact of embodiment on MI-BCI performance.

In conclusion, while prior virtual embodiment did not significantly alter MI training outcomes, the observed inter-individual variability suggests that embodiment effects may be more complex than previously assumed. These findings highlight the importance of designing adaptive and personalized VR-BCI protocols that account for individual differences in embodiment susceptibility. Future research should explore how factors such as training duration, sensory congruency, and neurophysiological predispositions influence the interaction between embodiment and MI-BCI performance.

## Data Availability

The raw data supporting the conclusions of this article will be made available by the authors, without undue reservation.

## Footnotes

^1^The online dataset used in this study is available at: https://zenodo.org/records/8086086.

^2^Video illustration of the experimental procedure is available at: https://www.youtube.com/watch?v=txPpFjRKIos.

^3^Source code and virtual environment used in the study are available at: https://github.com/noisys-project/Virtual-Embodiment-and-Motor-Imagery-BCIs.
